# Lead induces mouse skin fibroblast apoptosis by disrupting intracellular homeostasis

**DOI:** 10.1038/s41598-023-36835-5

**Published:** 2023-06-14

**Authors:** Hui Wang, Huinuan Wang, Jiawen Guan, Weijun Guan, Zheng Liu

**Affiliations:** 1 Jinzhou Medical University, Jinzhou, 121001 China; 2 Meat Processing and Safety Control Engineering Technology Research Center of Liaoning Province, Jinzhou, 121001 China; 3 Institute of Animal Science, Chinese Academy of Agricultural Sciences, Beijing, 100193 China

**Keywords:** Cell biology, Molecular biology

## Abstract

Lead (Pb) is a critical industrial and environmental contaminant that can cause pathophysiological changes in several cellular and organ systems and their processes, including cell proliferation, differentiation, apoptosis, and survival. The skin is readily exposed to and damaged by Pb, but the mechanisms through which Pb damages cells are not fully understood. We examined the apoptotic properties of Pb in mouse skin fibroblast (MSF) in vitro. Treatment of fibroblasts with 40, 80, and 160 μM Pb for 24 h revealed morphological alterations, DNA damage, enhanced caspase-3, -8, and -9 activities, and apoptotic cell population. Furthermore, apoptosis was dosage (0–160 μM) and time (12–48 h) dependent. Concentrations of intracellular calcium (Ca^2+^) and reactive oxygen species were increased, and the mitochondrial membrane potential was decreased in exposed cells. Cell cycle arrest was evident at the G0/G1 phase. The Bax, Fas, caspase-3 and -8, and p53 transcript levels were increased, whereas Bcl-2 gene expression was decreased. Based on our analysis, Pb triggers MSF apoptosis bydisrupting intracellular homeostasis. Our findings enrich the knowledge about the mechanistic function of Pb-induced cytotoxicity on human skin fibroblasts and could potentially guide future Pb health risk assessments.

## Introduction

Heavy metal pollution threatens human survival and development. Lead (Pb), one of the most common toxic heavy metals, is a ubiquitous environmental and occupational pollutant that can accumulate in the environment over a prolonged period^1^. Pb is widely used in batteries, paints, varnishes, pigments, and other daily-use products. Occupational Pb exposure, associated with dry cell manufacturing, mining activities, metallurgy, automotive machinery, and welding, has resulted in concerning Pb concentrations in air, water, soil, and food chains globally^2^. Pb has a long biological half-life and is non-degradable; hence, its excretion rate from the body is extremely slow. In the human body, once Pb is absorbed via inhalation, ingestion, or dermal contact, it accumulates in the bone, blood, and soft tissue, with a half-life of 20–30 years in bone and 35 days in blood^3^. Pb is a toxin that can cause various biochemical, physiological, and behavioral dysfunctions^4^. Pb exposure can induce pathological alterations in multiple organ systems, including the digestive^5^, hemopoietic^6^, urinary^7^, nervous^8^, immune^9^, respiratory^10^, reproductive^11^, circulatory^12^, endocrine^13^, and kinetic^14^ systems. Furthermore, Pb can cause apoptosis or necrosis of various cells in vitro, such as rat pheochromocytoma cells (PC12), rat retinal rod cells^15^, chicken liver cells^16^, rat hepatic and renal cells, and germ cells^17^.

As a widespread environmental toxin, Pb can exert a broad range of toxic effects on cells and organs. Pb can induce tissue damage and oxidative stress in animal models. Moreover, Pb can induce deleterious effects, such as reactive oxygen species (ROS) production, direct loss of antioxidant stores, cell membrane breakdown, enzymatic degradation, and DNA damage^18^. At the cellular level, Pb can reduce the mitochondrial membrane potential (MMP), consume the adenosine triphosphate (ATP) pool, and increase ROS production, resulting in ultrastructural and functional disturbances in mitochondrial metabolism^19^. In the early stage, Pb directly affects the expression of Fos and Jun and inhibits DNA repair, and indirectly plays a genotoxic role as a co-mutagen, causing chromatin compaction and aggregation, as well as impairing DNA and RNA synthesis^20^. Growing evidence has revealed that Pb can activate/inhibit several intracellular signaling pathways associated with cell survival and death^21^.

Typical apoptosis is mainly activated by two pathways, namely the extrinsic and intrinsic pathways. The extrinsic pathway is activated via death receptors on the cell surface and causes the activation of caspase-8 or -10, which initiates the caspase-3 cascade, leading to apoptosis. The endogenous pathway is divided into the mitochondrial and ER pathways; the mitochondrial pathway originates from the release of mitochondrial cytochrome C and is associated with the activation of caspase-9, which activates the downstream caspase-3 enzyme series and initiates the caspase cascade leading to apoptosis. The endoplasmic reticulum pathway results from the activation of apoptotic enzymes in the endoplasmic reticulum. Disruption of endoplasmic reticulum calcium homeostasis can activate caspase-12, and activated caspase-12 can further cleave caspase-3 and participate in cell apoptosis caused by the endoplasmic reticulum pathway.

Environmental Pb pollution seriously harms human health, and Pb can induce tissue and cell apoptosis through various pathways^22^. Apoptosis maintains homeostasis by removing excess and abnormal cells. Although the highest and most direct Pb exposure occurs through the skin, Pb-mediated induction of apoptosis in skin cells and the underlying mechanism have rarely been reported^23^. Pb-induced apoptosis is considered a possible mechanism of Pb toxicity^24^. Pb exposure-induced excessive or disordered apoptosis is a major factor in the development and spread of autoimmune and neurodegenerative neurological diseases^8^. Although the apoptotic potential of Pb has been well documented, it remains unclear whether Pb can induce apoptosis in skin fibroblasts. In the present study, mouse skin fibroblasts (MSFs) were incubated with various Pb concentrations to determine Pb-mediated toxic effects by analyzing the impact on cell survival, morphology, proliferation, and apoptosis. The cell cycle, MMP, intracellular Ca^2+^ homeostasis, ROS, caspase-3, -8, and -9 activities, and gene expression levels of Bax, Bcl-2, Fas, caspase-3 and -8, and p53 were analyzed to comprehensively clarify the toxicity mechanism of Pb on human skin fibroblasts and enrich the general knowledge of Pb toxicity mechanisms.

## Methods

### Materials

Herein, mice were not involved in the isolation of MSFs. We employed first to third passage MSFs from the animal genetic resources laboratory cell bank, Institute of Animal Science, Chinese Academy of Agricultural Sciences. The supplier authenticated the fibroblasts, which tested negative for contamination. Lead acetate (Pb(Ac)_2_; purity, 99.999%), 3-(4,5-dimethyl-2-thiazolyl)-2,5-diphenyl-2-H-tetrazolium bromide (MTT), propidium iodide (PI), acridine orange (AO), and ethidium bromide (EB) were acquired from Sigma Chemical Co. (St. Louis, MO). Annexin V-FITC Apoptosis Detection Kit I was obtained from Becton Dickinson Company (Becton Drive, Franklin Lakes, NJ). Dulbecco’s modified Eagle’s medium (DMEM) and fetal bovine serum (FBS) were purchased from Gibco (Gibco BRL Life Technologies, Grand Island, NY).

### Cell culture

MSFs were cultured in DMEM with 10% FBS under 5% CO_2_ and 95% air at 37 °C in a humid chamber. The medium was refreshed every 2 days. The cells were digested, dissociated, and divided into two sterilized flasks upon reaching 80–90% confluence.

### Growth dynamics

In total, 2 × 10^4^ cells/mL were seeded in a 24-well microporous plate. The cell growth rate was measured by counting three wells daily for 8 days using a hemocytometer. The growth curve was plotted, and the population doubling time (PDT) was assessed depending on the growth curve.

### Drug preparation and cell exposure

Pb^2+^ was provided to cells as Pb(Ac)_2_, dissolved and diluted to the desired concentration with DMEM, and filtered for sterilization. Upon entering the logarithmic growth phase, cells were exposed to multiple Pb^2+^ concentrations (0, 40, 80, and 160 μM) for prespecified durations (12, 24, 36, and 48 h).

### Cell survival assay

Cell survival was evaluated via MTT assay. In total, 1 × 10^5^ cells/well cell suspensions (200 μL) were added to 96-well microplates. The old medium was removed, and 180 μL of fresh complete medium with varying Pb^2+^ concentrations was added when cells entered the logarithmic phase. Simultaneously, corresponding controls and replicates were established. Cells were cultured for 12, 24, 36, and 48 h. MTT solution (20 μL, 5 mg/mL) was introduced, and cells were incubated for 4 h. The supernatant was removed, and the precipitate was resuspended in dimethyl sulfoxide (DMSO; 200 μL). Absorbance was detected at 490 nm using a microplate reader (Elx 800; BioTek Instruments, Winooski, VT, USA).

### AO/EB double staining

Cells were exposed to Pb^2+^ for 24 h, and cell suspensions were collected prior to staining with AO and EB solutions (2 mg/mL in ethanol). The cells were incubated at 25 °C in the absence of light for 6 min, followed by imaging with a confocal microscope (Nikon TE-2000-E, Tokyo, Japan).

### Hoechst 33258 staining

Briefly, coverslips were sterilized and added to culture plates. Cells were allowed to grow on coverslips, treated with Pb^2+^ for 24 h, and subsequently entered the logarithmic growth phase. The control group was established simultaneously. After discarding the culture medium, a fixative was introduced prior to rinsing and staining with Hoechst 33258. Anti-fluorescence quenching sealing solution was dripped onto the glass slide, and coverslips with cells were covered on the glass slide, ensuring that cells were fully in contact with the sealing solution while avoiding bubble formation. Cells were observed and photographed under a confocal microscope.

### Comet assay

Upon cell harvest, cells were rinsed in cold phosphate-buffered saline (PBS), and 1 × 10^5^ cells/mL was suspended in PBS prior to the comet assay as described in the Alkaline Comet Assay kit protocol (Trevigen Inc., Gaithersburg, MD, USA). The cell suspension was combined with low-melting-point agarose, and the samples were transferred with a pipette onto comet slides prior to storage in ice-cold lysis buffer (Trevigen Inc.) at 4 °C for 35 min. The comet slides were electrophoresed with alkaline electrophoresis buffer and treated with 70% ethanol for 6 min before drying. DNA was stained with SYBR green I (Sigma) and photographed under a confocal microscope.

### Transmission electron microscopy (TEM)

The cells were collected, rinsed twice with PBS, fixed in 2.5% glutaraldehyde and then in 1% osmium tetroxide, and rinsed using 0.1 mol/L of PBS. An ethanol series (30%, 50%, 70%, 80%, 90%, and 100%) was used for cell dehydration, followed by cell embedding in an epoxy resin using acetone, which was left to solidify. Ultrathin sections (50 nm) of the resin were cut using an ultrathin microtome (LEICAUC6i) and stained using uranyl acetate and lead citrate prior to imaging using a transmission electron microscope (JEM-2000Ex; JEOL Ltd., Tokyo, Japan).

### Annexin V‑fluorescein isothiocyanate (FITC)/PI double‑labeling

Apoptosis was assessed based on the Annexin V‑FITC Apoptosis Detection kit directions. Upon harvest, cells were diluted to 5 × 10^5^ cells/mL with binding buffer. Next, 5 μL each of PI and FITC were introduced to each 100 μL cell suspension sample, followed by incubation in 400 μL binding buffer for 10 min. Cells were analyzed via flow cytometry (FC500, Beckman Coulter, Brea, CA). A minimum of 10,000 cells were assessed per sample.

### Cell cycle analysis

Upon harvest, cells were resuspended in cold 70% ethanol, incubated at 4 °C for 12 h, rinsed in PBS, incubated in RNase A (0.02 mg/mL), and stained with PI solution (sodium citrate: 1 mg/mL, PI: 0.05 mg/mL, NaCl: 0.585 g/mL, pH: 7.2–7.6) for 30 min at 4 °C. Cells were analyzed via flow cytometry. A minimum of 10,000 cells were assessed per sample.

### MMP

Following harvest, cells were diluted to 1 × 10^6^ cells/mL; 0.5 mL 5,5′,6,6′-tetrachloro-1,1′,3,3′-tetraethylbenzimidazolcarbocyanine iodide (JC-1) working solution (5 mg/mL) was introduced to each sample and then maintained in a CO_2_ incubator for 10 min, followed by twice rinsing with prewarmed PBS. Cells were resuspended in 0.5 mL PBS before analysis via flow cytometry. Overall, 10,000 cells were assessed per sample.

### Intracellular Ca^2+^ homeostasis

DMSO was used to dissolve Fluo3-AM (Invitrogen, Carlsbad, CA, USA) to form a 2 mM solution before cryogenization at − 70 °C. Following harvest, cells were diluted to 1 × 10^6^–2 × 10^6^ cells/mL using serum-free culture media and loaded with Fluo3-AM (final concentration of 5 μM). Negative controls (without adding Fluo3-AM) were prepared prior to incubation under 5% CO_2_ at 37 °C without light for 35 min. Images were captured under a confocal microscope.

### ROS analysis

Cells were harvested, concentration-adjusted to 1 × 10^6^–2 × 10^6^ cells/mL, attached to 2′,7′-dichlorodihydrofluorescein-diacetate (DCFH‑DA) (Molecular Probes, Eugene, OR, USA) to a final concentration of 8 μM, prior to incubation at 37 °C under 5% CO_2_ for 15 min. Images were captured under a confocal microscope.

### Caspase-3, -8, and -9 activity assay

Caspase‑3, ‑8, and ‑9 activities were analyzed using a caspase activity kit (Beyotime Institute of Biotechnology, Haimen, China) based on kit directions and as reported before^25^.

### Real-time quantitative polymerase chain reaction (qPCR)

Total RNA was isolated, and complementary DNA (cDNA) was synthesized with a Primer Script TM RT kit (Takara, Dalian, China). Targeted GAPDH (housekeeping gene), Bcl-2, Bax, Fas, caspase-3, caspase-8, and p53 primers were prepared using the Primer Premier 5.0 software (PREMIER Biosoft, Palo Alto, CA, USA) and generated by Shanghai Biotechnology Co. Ltd (Shanghai, PR China). qPCR was performed using an ABI 7500 qPCR system (Applied Biosystems, Foster City, CA, USA) and qPCR Detection Kit (Takara) as per kit instructions under the following conditions: denaturation (94 °C, 3 min), denaturation (40 cycles, 94 °C, 30 s), annealing (52–55 °C, 30 s), and elongation (72 °C, 30 s). Each experiment was repeated three times and performed in duplicate in 96-well plates. Relative gene levels were computed using the 2^−△△Ct^ formula, and alterations in gene levels were evaluated via the Student’s t-test (*P* < 0.05 was set as the significance threshold).

### Statistical analysis

All experiments were performed in triplicate to verify reproducibility. Data are presented as mean ± standard deviation (SD), and analysis was carried out using the Statistical Analysis System (SAS) software (SAS Institute Inc., Cary, NC, USA) and comparison via a multiple comparison test (DUNCAN). *P* < 0.05 was set as the significance threshold.

## Results

### Growth dynamics

**Figure 1:**
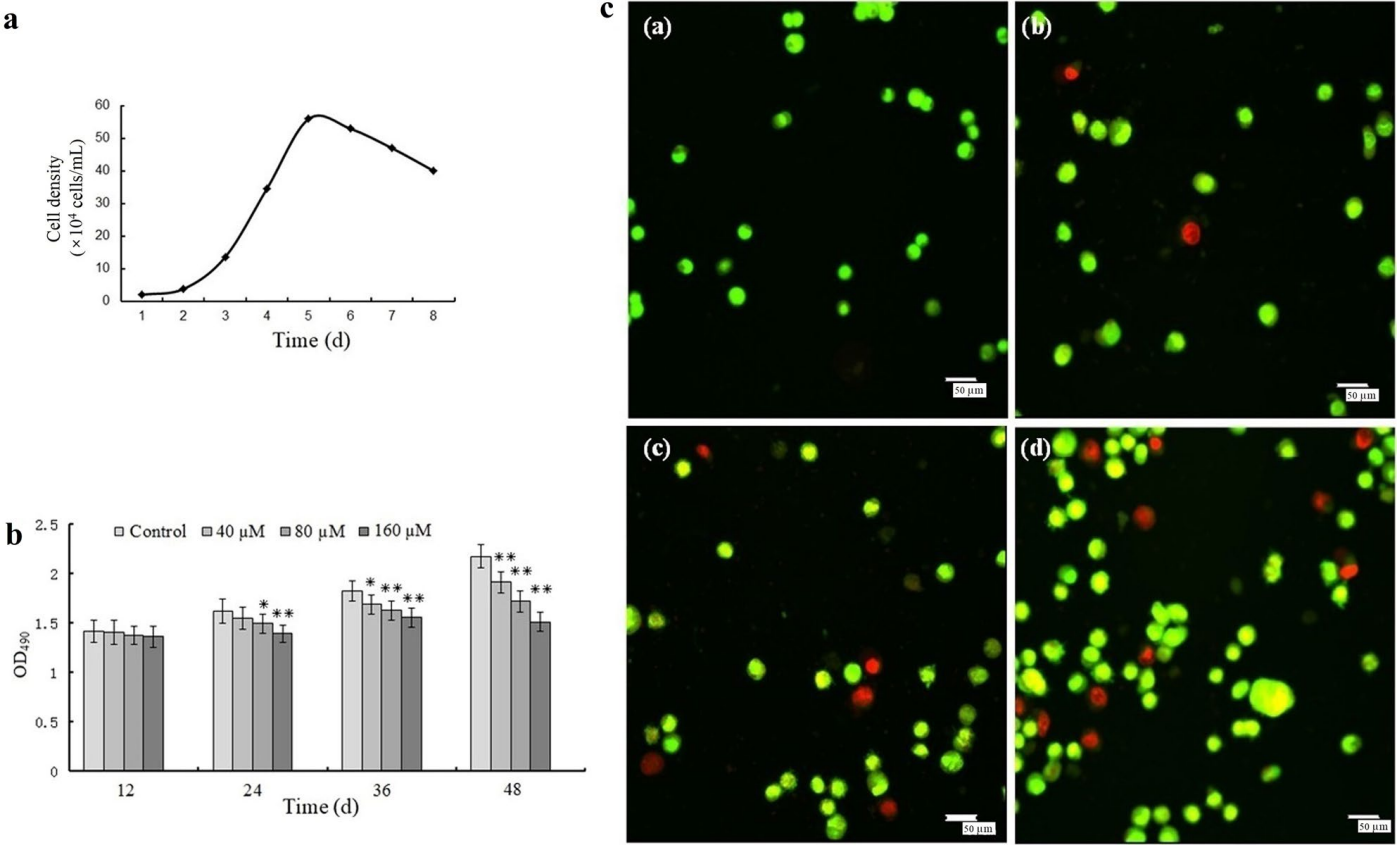
Growth dynamics, cytotoxicity analysis, and AO/EB double staining. (**a**) Mouse skin fibroblast (MSF) growth curve. (**b**) Cytotoxicity assessment. c Morphological evaluation of nuclear damage in MSFs after Pb^2+^ exposure for 24 h. (**a**) Control, (**b**) 40 μM, (**c**) 80 μM, and (**d**) 160 μM; scale bar = 50 μm. Statistically different values relative to matched controls are labeled with * (*P* < 0.05) and ** (*P* < 0.01; n = 3).

The MSF growth curve presented a strong “S” outline, and the PDT was approximately 32 h. The cells entered a latent phase from days 1–2, entering the logarithmic phase from days 2–5. The density peaked on day 5, reached a plateau on day 6, and subsequently declined (Fig. 1a).

### Cell survival assay

The MTT assay was utilized to determine the mitochondrial succinate dehydrogenase activity and detect mitochondrial function, which is widely used to evaluate cell viability. Pb^2+^ reduces the viability of MSFs. Cell survival decreased considerably with increased Pb^2+^ concentration and duration of exposure; in other words, cell survival reduced in a dose‑ and time‑dependent manner (Fig. 1b).

### AO/EB double staining

In normal cells, the nuclear chromatin is pyknotic or beaded. The 40 µM treatment group showed numerous early apoptotic cells with pale yellow nuclei. In the 80 µM treatment group, some cells exhibited uneven morphology and late apoptosis. The 160 µM treatment group exhibited a considerable number of late apoptotic cells, with orange-red nucleosomes, concentrated chromatin, variable sizes, and relatively thick staining. Some late apoptotic cells were close to death (Fig. 1c).

### Hoechst 33258 staining

**Figure 2:**
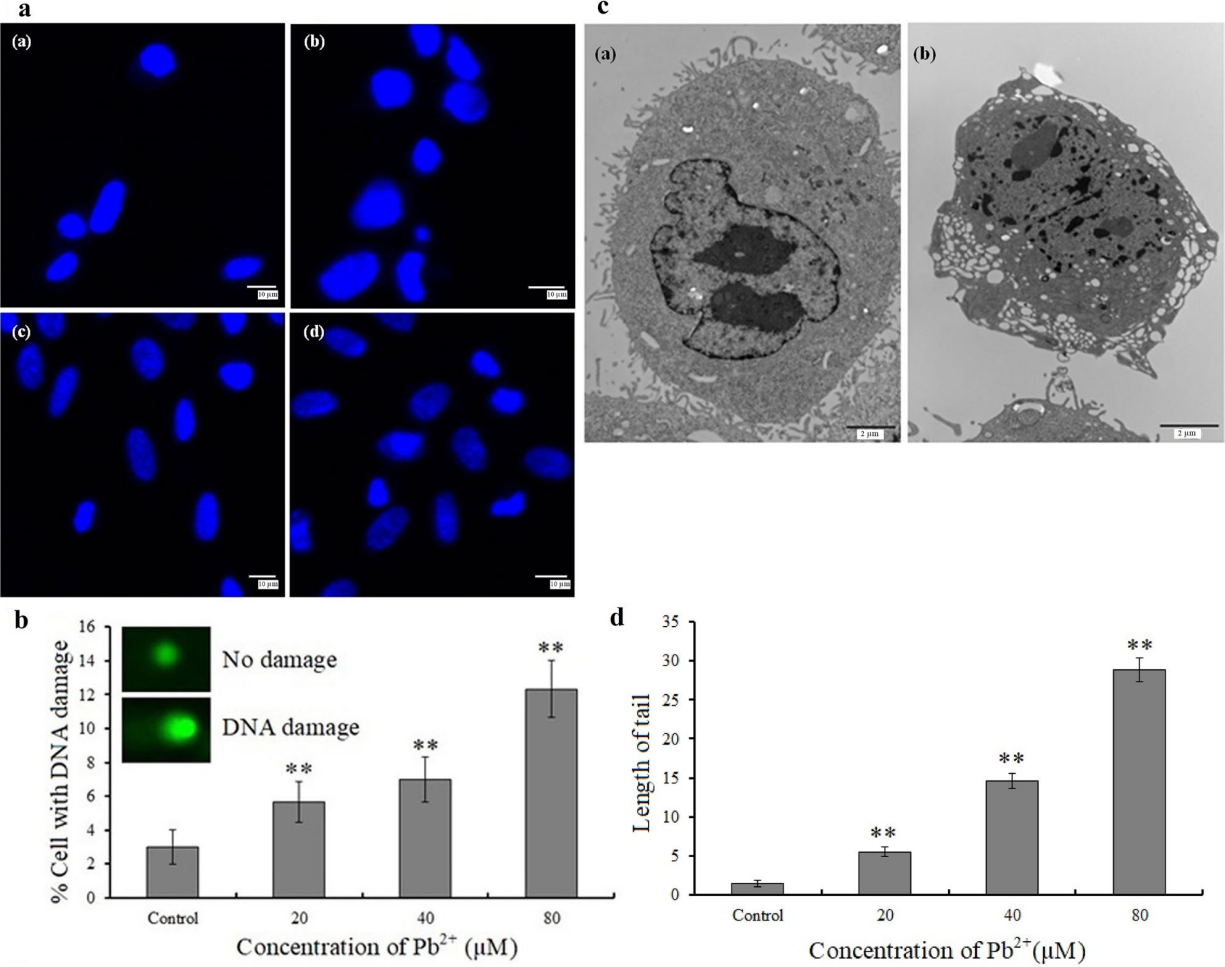
Hoechst 33258 staining, comet assays, and transmission electron microscopy (TEM). (**a**) Morphological evaluation of mouse skin fibroblasts (MSFs) 24 h after Pb^2+^ exposure using Hoechst 33258 staining by confocal microscopy. (**a**) Control, (**b**) 40 μM, (**c**) 80 μM, and (**d**) 160 μM; scale bar = 10 μm. (**b**) and (**d**) Comet assay showed DNA damage in MSF exposed to Pb^2+^ for 24 h. Significantly different values relative to matched controls are labeled with ** (*P* < 0.01; n = 3). (**c**) Subcellular assessment via TEM. (**a**) Control and (**b**) 160 μM; scale bar = 2 μm.

The nuclear chromatin of control cells was uniformly stained, displaying round or ovoid nuclei, and emitting strong fluorescence. The treatment group showed cell chromatin concentration, uneven coloring, nuclear pyknosis, and bright spots, with nuclear pyknosis worsening with increasing Pb^2+^ concentration (Fig. 2a).

### Comet assay

To analyze DNA damage, 100 fibroblasts were randomly selected from each group. The DNA of control cells was confined to the nucleus, while DNA strands of Pb^2+^-treated cells were broken, with DNA fragments traveling along the electric field, forming a bright fluorescent trail (Fig. 2b). The trail size indicated injury severity, which increased with increasing Pb^2+^ doses (Fig. 2d), thereby indicating that Pb^2+^ could disrupt the DNA integrity of MSFs.

### TEM

The control cells exhibited abundant cell surface microvilli, uniform cytoplasm, intact nuclear membrane structure, clear and complete nucleoli, and uniform staining. Considering Pb^2+^-treated cells, cell surface microvilli disappeared, vacuoles appeared in the cytoplasm, and the nuclear membrane structure was damaged. The chromatin of apoptotic cells exhibited chromatin was condensed, marginalized, and segmented into clumps (Fig. 2c).

### Annexin V-fluorescein isothiocyanate (FITC)/PI double labeling

**Figure 3:**
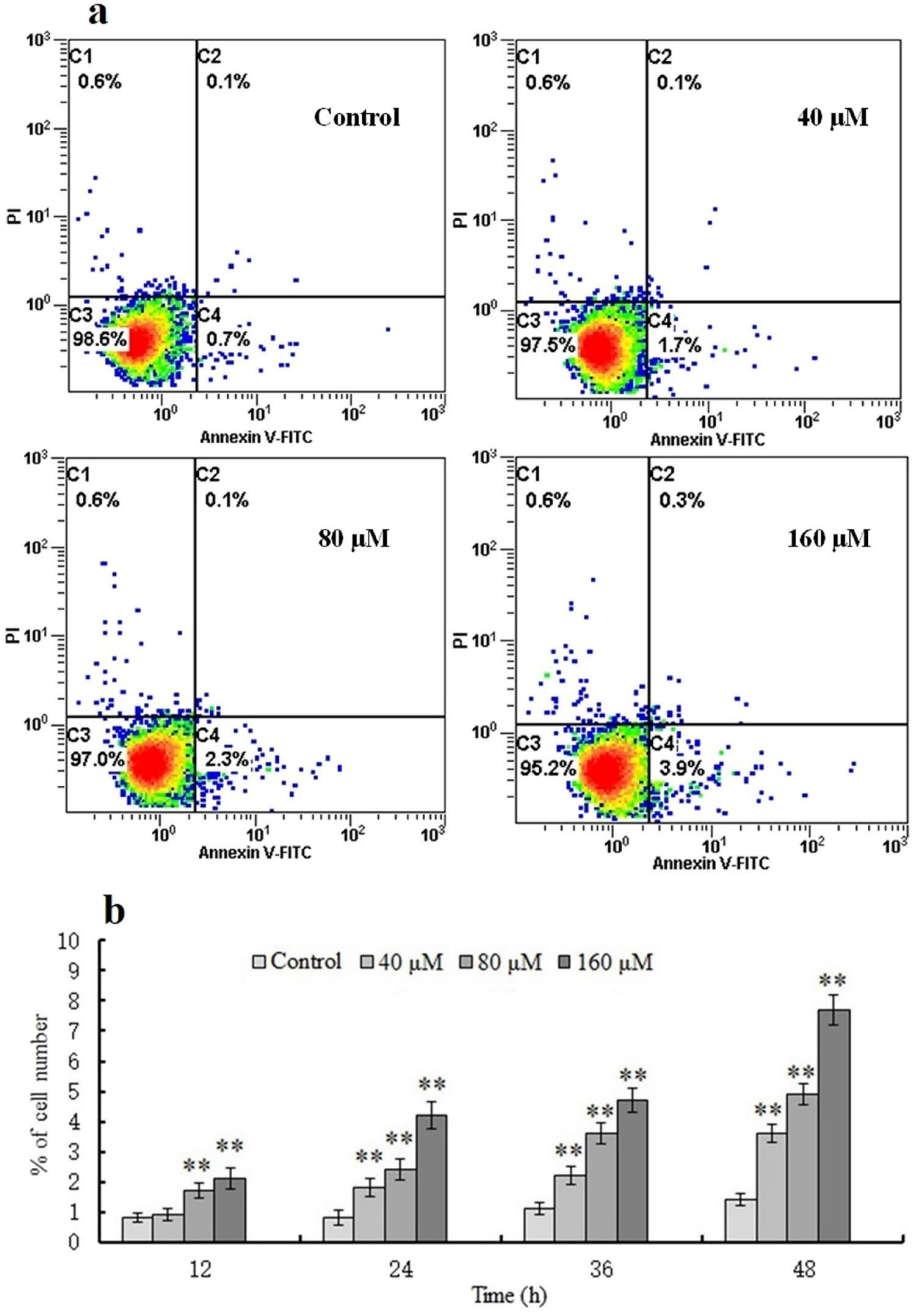
Annexin V-FITC/PI double labeling. (**a**) Scatter plot of the apoptotic rates of mouse skin fibroblasts (MSFs) treated with 0, 40, 80, or 160 μM Pb^2+^ for 24 h (12, 36, and 48 h plots not shown). (**b**) Apoptotic rates of MSFs exposed to 0, 40, 80, or 160 μM Pb^2+^ for 12, 24, 36, and 48 h. Significantly different values relative to matched controls are labeled with ** (*P* < 0.01; n = 3).

Following exposure of MSFs to 0, 40, 80, and 160 μM of Pb^2+^ for 12 to 48 h, the apoptotic rate (*P* < 0.01) significantly increased from 0.4 to 9.7% when compared with that of the control group (12, 36, and 48 h; histogram plots not shown; Fig. 3a). Furthermore, apoptosis was dose- and time-dependent (Fig. 3b).

### Cell cycle analysis

**Figure 4:**
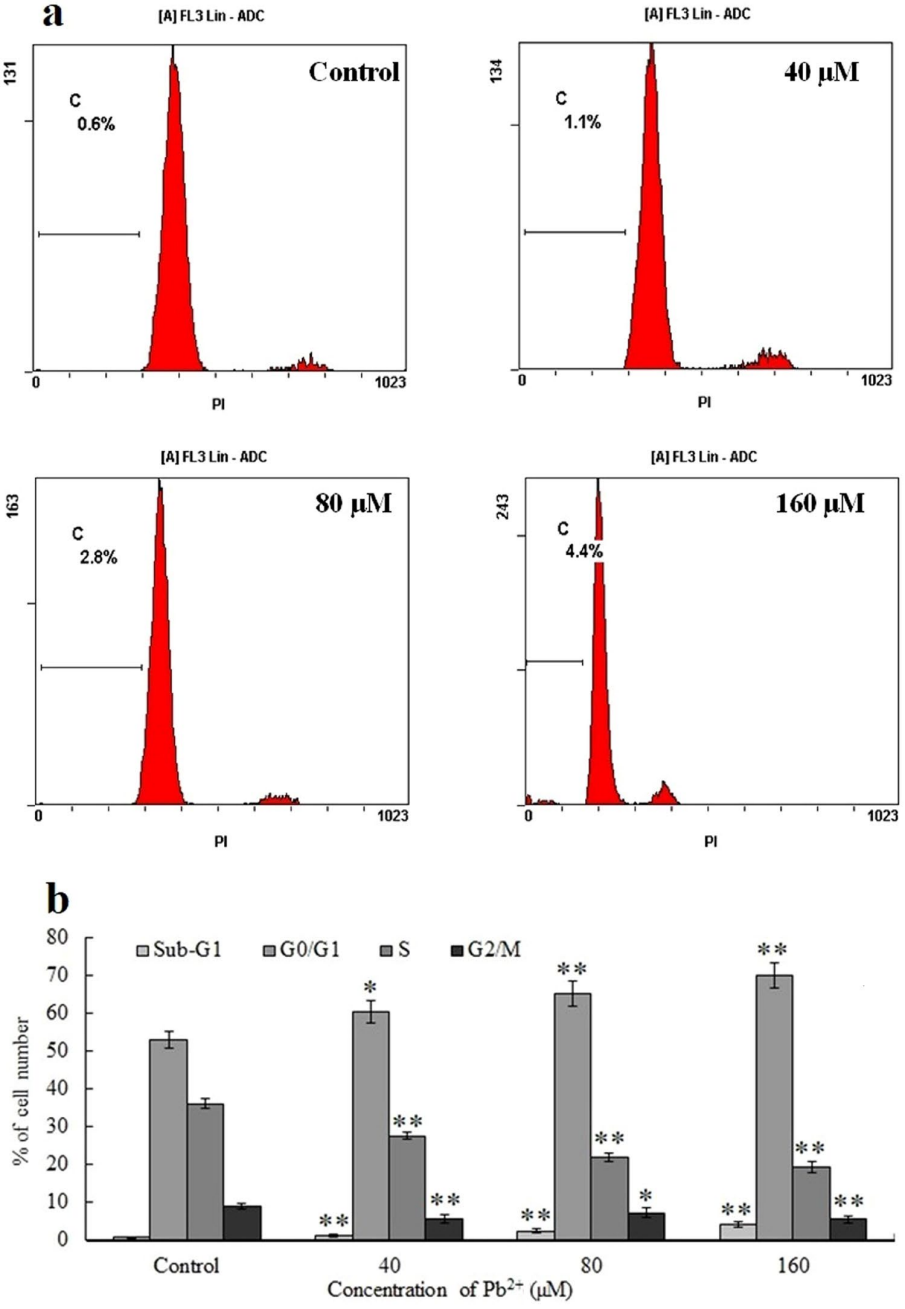
Cell cycle progression. (**a**) Cell cycle progression and sub-G1 levels in mouse skin fibroblasts (MSFs) following Pb^2+^ exposure for 24 h. (**b**) Cell distribution in the sub-G1, G0/G1, S, and G2/M cell cycle phases. Significantly different values relative to matched controls are labeled with * (*P* < 0.05) and ** (*P* < 0.01; n = 3).

With increasing Pb^2+^ concentration, the proportion of G0/G1 cells increased, and the proportion of S and G2 phase cells decreased accordingly. Based on these results, there was a dose-dependent association between Pb^2+^ levels and cell cycle dysregulation, indicating that Pb^2+^ inhibited DNA synthesis in the G0/G1 phase (Fig. 4a). In the diploid DNA peak (sub-G1 population), the proportion of apoptotic cells increased in a dose-dependent fashion (Fig. 4b).

### MMP

**Figure 5:**
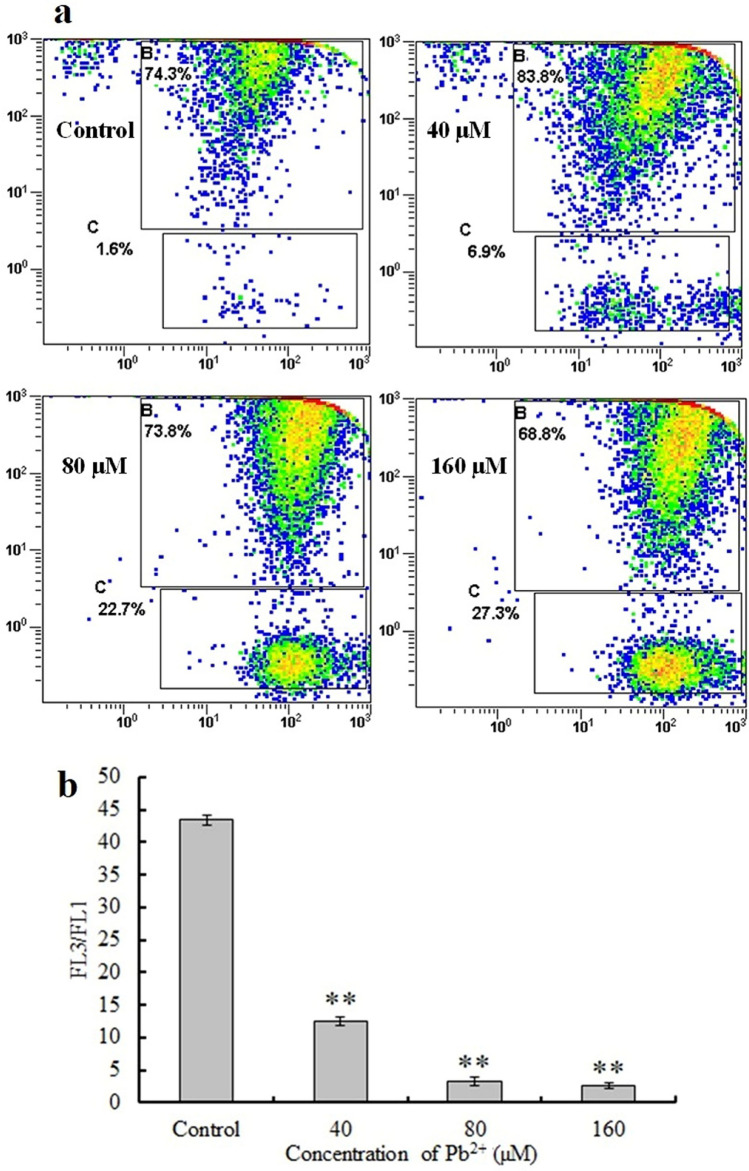
Mitochondrial membrane potential (MMP). (**a**) Scatter plot of mouse skin fibroblast (MSF) MMP following treatment with 0, 40, 80, or 160 μM Pb^2+^ for 24 h. (**b**) The ratio of fluorescence intensity. Significantly different values relative to matched controls are labeled with ** (*P* < 0.01; n = 3).

JC-1 is a lipophilic cationic stain that exclusively enters mitochondria and reversibly changes it from a green to a red color with increasing MMP. In normal cells, JC-1 accumulates in the mitochondria and shows red fluorescence. In apoptotic cells, JC-1 exists as a monomer with green cytoplasm, indicating a low MMP. The proportion of C-gated cells indicates the alterations in the MMP; a larger quantity represents a decrease in the MMP (Fig. 5a). Accordingly, the MMP of treated cells was significantly reduced when compared with that of control cells (*P* < 0.01) (Fig. 5b).

### Intracellular Ca^2+^ homeostasis

**Figure 6:**
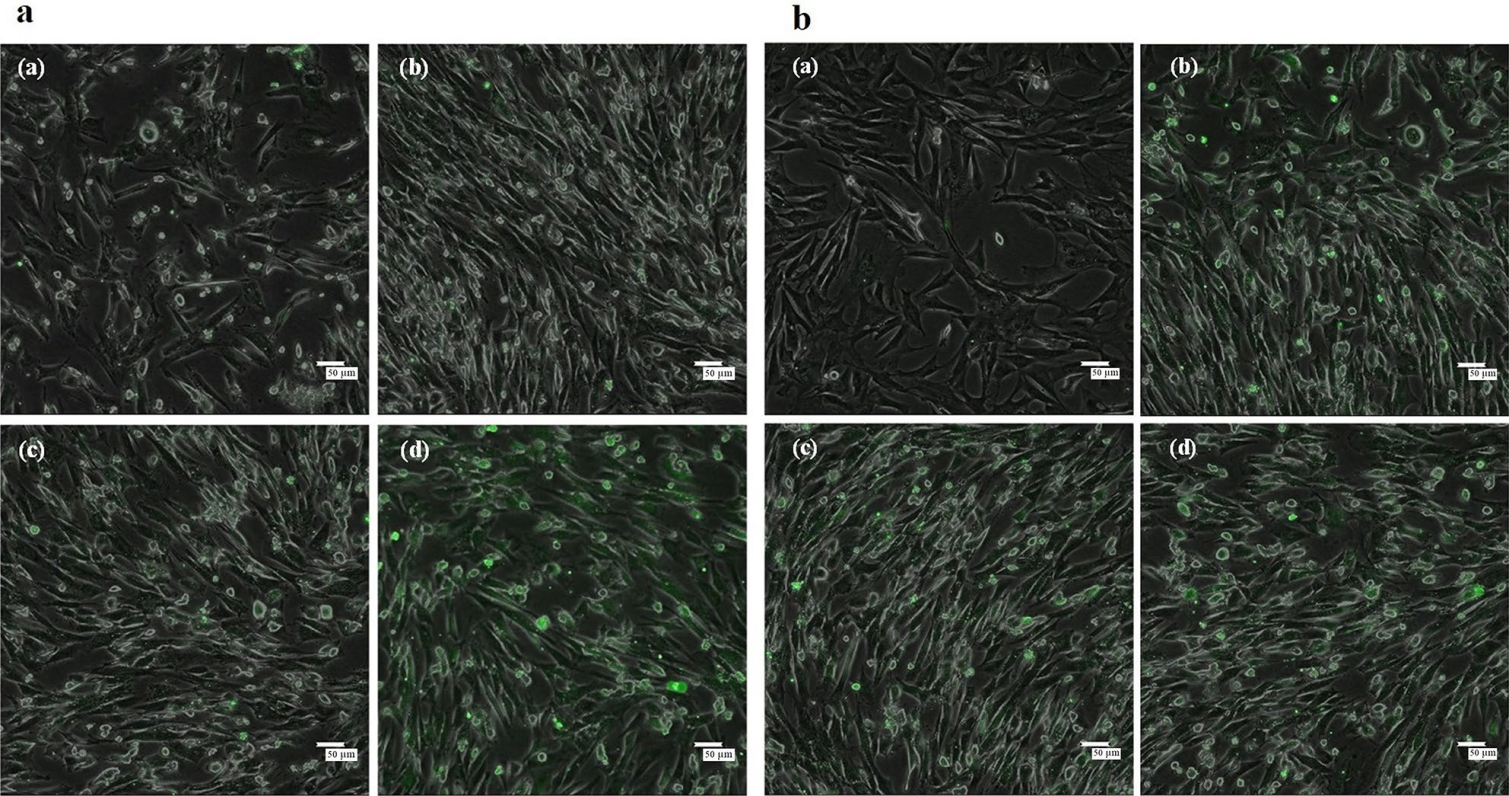
Intracellular Ca^2+^ homeostasis and ROS analysis. (**a**) Effect of Pb^2+^ exposure on intracellular Ca^2+^ homeostasis in mouse skin fibroblasts (MSFs) after treatment for 24 h. (**b**) Effect of Pb^2+^ exposure on intracellular ROS levels in MSFs after treatment for 24 h. Both (**a**) and (**b**) show (**a**) Control, (**b**) 40 μM, (**c**) 80 μM, and (**d**) 160 μM; scale bar = 50 μm. ROS, reactive oxygen species.

Upon treatment with higher Pb^2+^ concentrations, apoptotic cells showed a higher proportion and intensity of green fluorescence, indicating an increase in free Ca^2+^ levels. The green fluorescence observed in 160 μM Pb^2+^-treated cells was evenly distributed, suggesting a severe disruption in intracellular Ca^2+^ homeostasis (Fig. 6a).

### ROS analysis

The higher the Pb^2+^ concentration, the greater the number of apoptotic cells and the stronger the green fluorescence (Fig. 6b). The green fluorescence intensity increased, suggesting that ROS concentration also increased. The green fluorescence was distributed throughout the cells in the 80 and 160 μM Pb2 + treatment groups.

### Caspase-3, -8, and -9 activity analyses

**Figure 7:**
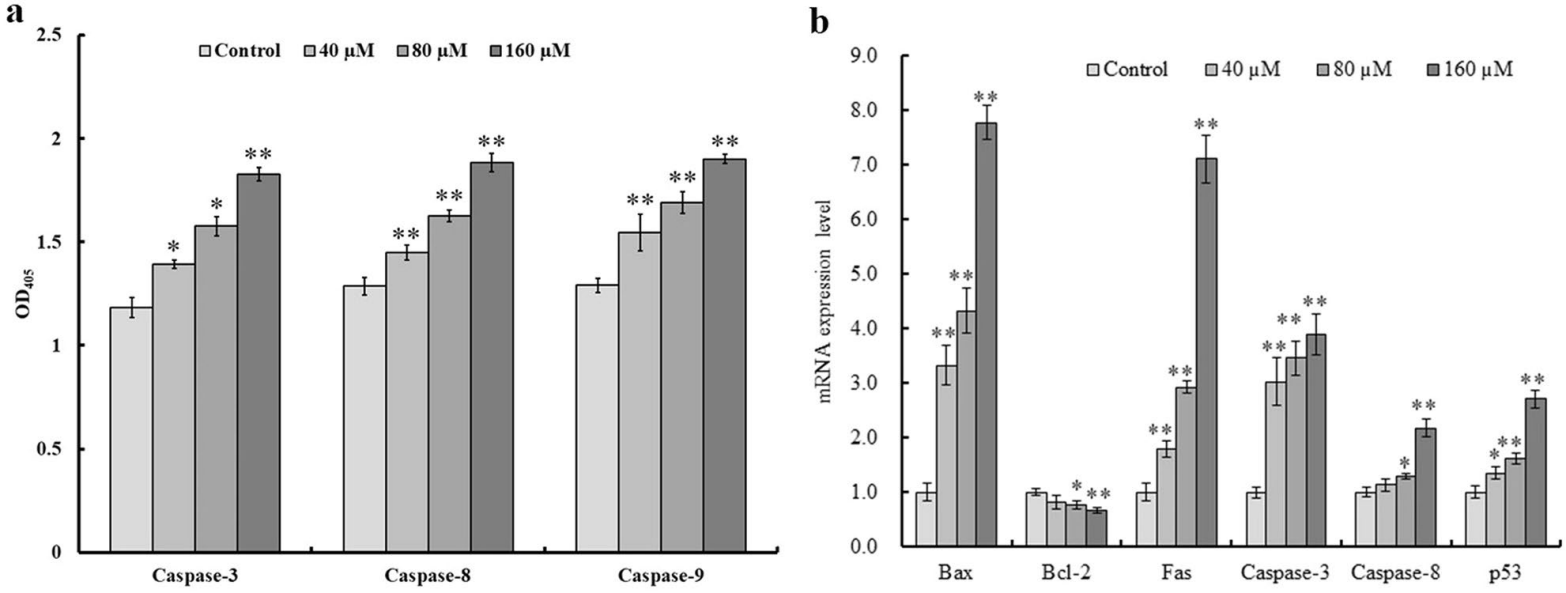
Caspase-3, -8, and -9 activity assays and gene expression levels. (**a**) Caspase-3, -8, and -9 activities were assessed in cytosolic extracts of mouse skin fibroblasts (MSFs) after Pb^2+^ treatment for 24 h. (**b**) Evaluation of Bax, Bcl-2, Fas, caspase-3, caspase-8, and p53 transcript expression at 24 h (adjusted to GAPDH). Significantly different values relative to matched controls are labeled with * (*P* < 0.05) and ** (*P* < 0.01; n = 3), which applies to (**a**) and (**b**).

Caspase-3, -8, and -9 are critical for cell apoptosis, serving as active proteases. Based on our analysis, the caspase-3 activity differed significantly in the 40 and 80 μM Pb^2+^ treated cells when compared with that in control cells (*P* < 0.05). The cell number was significantly increased in the 160 μM Pb^2+^-treated group when compared with that in the control group (*P* < 0.01). Caspase-8 and -9 activities notably differed in cells of all three treatment groups when compared with those in control cells (*P* < 0.01, Fig. 7a). The caspase-3, -8, and -9 activities increased with increasing Pb^2+^ concentrations in a dose-dependent fashion.

### qPCR

Considering glyceraldehyde-3-phosphate dehydrogenase (GAPDH) as the endogenous control, Bax and Bcl-2 are expressed in the outer mitochondrial membrane, and the Bax/Bcl-2 ratio strongly regulates apoptosis. Based on the qPCR analysis, treatment with 160 µM Pb^2+^ significantly increased Bax, Fas, caspase-3, caspase-8, and p53 levels in mouse fibroblasts (*P* < 0.01) but significantly decreased Bcl-2 expression (*P* < 0.01, Fig. 7b). these results indicate that Pb^2+^ treatment increased the Bax/Bcl-2 ratio in mouse fibroblasts.

## Discussion

Environmental pollution attributed to heavy metals caused by human activities is a global concern. Pb, a heavy metal responsible for environmental pollution, is a non-biodegradable inorganic pollutant widely distributed in soil–water–air ecosystems owing to its easy migration and transformation^26^. Pb can cause a series of behavioral, biochemical, and physiological dysfunctions in organisms^27^. Toxicological and epidemiological studies have documented Pb-induced toxicity in several cells, organs, and organ systems^28^. Pb accumulation in living organisms has deleterious long-term effects, given that Pb induces cytotoxicity in various cell types through distinct cytotoxic pathways^29^. For instance, Pb suppresses the proliferation of human leukemia cells by inducing cell cycle arrest, DNA damage, and accelerating apoptosis via caspase-3 activation^30^. Pb mediates mitochondrial permeability transition pore (MPTP) opening, releases mitochondrial cytochrome c, and induces apoptosis of proximal tubule cells. Furthermore, Pb can induce apoptosis of rat proximal tubular cells through endoplasmic reticulum stress^31^. The skin is one of the major target organs for Pb poisoning. However, the Pb toxicity pathway in the skin remains elusive. Herein, we present evidence to suggest that Pb exposure suppresses MSF cell survival and promotes cell apoptosis.

Pb has been shown to significantly reduce TM3 cell proliferation in a concentration- and time/dependent manner^29^. Following treatment of HK-2 cells with increasing concentrations of lead nitrate (0–50 µM) for 24 h, intracellular ROS levels increased, whereas glutathione (GSH) levels decreased substantially in a dose-dependent manner^32^. Our findings are consistent with prior toxicological investigations involving other cell lines, showing a dose-dependent decrease in MSF survival, as evidenced by the MTT assay and Annexin V-FITC/PI double labeling. Annexin V- FITC/PI double labeling showed that Pb strongly promoted apoptosis, and the apoptotic-promoting effect was more notable with increasing Pb concentrations. AO/EB staining, Hoechst 33258 staining, and comet assay were used to demonstrate that Pb could induce MSF apoptosis. The membrane permeability of early apoptotic cells was relatively low, and ethidium bromide did not easily enter the cell interior; the cells were light yellow, with pyknotic or bead-like nuclear chromatin. The membrane permeability of late apoptotic cells was higher than that of early-stage apoptotic cells, but differed in terms of membrane rupture, with cells appearing orange-red and relatively more stained; some late apoptotic cells were close to death to a certain extent (Fig. 1c). Our assay results indicated that Pb could induce DNA damage, which is a unique change in apoptotic cells. The comet assay is a sensitive method for assessing individual cell nucleosomal DNA fragmentation. Ultrastructure analysis by electron microscopy is considered the optimal approach for identifying cell apoptosis. Based on our TEM analysis, Pb could induce typical apoptotic morphological changes, including cell shrinking, mitochondrial depolarization, subcellular structure destruction, and chromatin condensation in MSFs. In addition, flow cytometric analysis revealed that Pb treatment considerably increased the proportion of apoptotic cells in a concentration-dependent manner. Our results are consistent with those of previous studies, in which Pb accelerated apoptosis in TM3 cells^33^, cardiomyocytes^34^, and human monocyte-macrophage (THP-1) cells^35^.

Apoptosis, gene-controlled programmed cell death, is crucial for maintaining the internal environmental balance and normal body development. The cell cycle is a highly regulated process that modulates cell development and differentiation^36^. Given that apoptosis stimulation affects the cell cycle, alterations in cell cycle distribution may be related to apoptosis and differentiation. Hence, cell cycle analysis is among the most informative assessments for determining apoptosis. Pb was found to strongly trigger cell cycle arrest in the G0/G1 phase in the HL-60 cell lines^30^. To verify whether the Pb-mediated MSF suppression is related to cell cycle arrest, we examined cell cycle progression using flow cytometry. Based on our analysis, Pb caused G0/G1 phase cycle arrest in MSFs; the proportion of G1 cells and the level of hypodiploid DNA (sub-G1 population) were increased, the amount of S-phase cells was decreased, and cell proliferation was inhibited. Therefore, we hypothesized that Pb interferes with DNA replication and cell cycle distribution, resulting in MSF apoptosis. Mitochondria are necessary for apoptosis and are considered central regulators of apoptosis in vertebrates. One early event of apoptosis involves a reduction in MMP past a particular point. Pb reportedly binds to mitochondria at ion sites, resulting in a decrease in the membrane potential, shifting mitochondrial channels to an active open state^37^. Therefore, cell apoptosis is initiated when mitochondrial polarization, swelling, and membrane rupture occur along with the release of cytochrome C and other cytokines into the cytoplasm^38^. Pb treatment can stimulate the mitochondria-based apoptotic network^39^. Pb-driven mitochondria-based primary cultured rat proximal tubular cell apoptosis was shown to depend on the opening of the MPTP, and the three regulatory components of MPTP (CYPD, ANT, and VDAC) play roles in Pb-linked MPTP opening^34^. We demonstrated that the MMP of MSFs decreased dose-dependently with increased Pb exposure, indicating that Pb-driven MSF apoptosis is associated with mitochondria-based apoptosis. The Pb-based toxic sites in the mitochondria and the relative sensitivity of multiple mitochondria-mediated pathways remain poorly elucidated.

Under normal conditions, the body has a certain amount of ROS. As the messenger of various metabolic and signaling pathways within the body, ROS participates in the transcription of various genes and the expression of related functional proteins by activating and regulating various transcription factors, participating in cell proliferation, differentiation and maintenance of cells and tissues, and body metabolism^40^. ROS has strong reactive activity and oxidation. High ROS concentrations can directly or indirectly cause oxidative damage to lipid substances such as DNA, protein and cell membranes and induce cell apoptosis, senescence, and death^41^. In mouse renal proximal tubular cells as target cells, Pb exposure caused cell apoptosis, necrosis, mitochondrial damage and other cell damage, accompanied by increased intracellular ROS content^42^. Long-term low-dose Pb exposure can impact the structure and function of mouse renal proximal tubule cells. In addition, Pb exposure was found to alter cell morphology and hinder the ability of cells to take up calcium ions. These physiological changes are related to the content of intracellular ROS and occur in a dose-dependent manner^43^. Low concentration, acute Pb exposure could activate the activity of protein kinase C (PKC) in cells, which, in turn, regulated the calcium transport related N-methyl-d-aspartic acid receptor (NMDA) receptor channel, thereby increasing intracellular Ca^2+^ content. However, NMDA receptor activation will promote the release of excitatory amino acid glutamate, subsequently triggering an increase in intracellular ROS content, resulting in oxidative stress^44^. Oxidative stress and cell apoptosis are the two main pathways through which Pb exerts its toxic effects. Pb exposure-induced oxidative stress can accelerate the process of cell apoptosis, as well as impact ROS production, consequently affecting the process of oxidative stress^45^.

Ca^2+^, a secondary messenger, is necessary for cell metabolism; an imbalance in Ca^2+^ homeostasis can induce apoptosis through various pathways. The initial increase in Ca^2+^ may be attributed to the influx of Ca^2+^ in the extracellular space or redistribution of intracellular storage, resulting in irreversible or reversible changes in several major pathways^46^. Increased intracellular free Ca^2+^ activates multiple signals and events involved in almost all aspects of cellular activity, ultimately inducing apoptosis^47^. Based on our analysis, Pb treatment increased cytoplasmic free Ca^2+^ concentration, disrupting intracellular Ca^2+^ homeostasis. In workers exposed to Pb, eryptosis associated with oxidative damage seems to be characterized by a rise in free intracellular Ca^2+^, owing to enhanced Ca^2+^ channel permeability and diminished Ca^2+^ Mg^2+^ (ATPase) activity^48^.

Apoptosis may be related to an imbalance in calcium homeostasis, oxidative damage, and mitochondrial damage^49^. Under physiological conditions, the production and clearance of oxygen free radicals are in a dynamic equilibrium. Several factors may reduce the ability of the antioxidant system to scavenge oxygen free radicals, thereby upregulating oxygen free radicals. Pb exposure can induce the generation of oxygen free radicals, and oxidative stress directly damages cell membranes, leading to increased cell membrane permeability^50^. Herein, Pb treatment resulted in increased ROS levels in MSFs. Our results corroborate the findings of a previous study^50^ and suggest that Pb can induce oxidative stress in MSFs in vitro. Excessive ROS production has consistently been shown to induce apoptosis.

Caspases are a family of proteases, and the caspase-induced cascade is critical for the stimuli-induced apoptotic process. Caspase-3 is primarily activated by cytotoxic drugs^51^, and it strongly regulates apoptotic DNA damage. In human leukemia cells, Pb was shown to modulate apoptosis via caspase-3 activation^30^. Herein, Pb-exposed MSFs showed increased caspase-3, -8, and -9 activities. Our results corroborate those of a previous study, which demonstrated that Pb exposure could promote renal cell apoptosis and impair cell function by activating caspase-3^52^.

Bcl-2 is a major contributor to cell death signaling, and the protein family has both anti- and pro-apoptotic members. Bcl-2 regulates the balance between pro-apoptotic members like Bax and pro-survival Bcl-2-like proteins and functions as an anti-apoptotic protein that modulates multiple apoptotic networks. The Bax protein is considered to be oligomeric and can enhance mitochondrial outer membrane permeability^53^. A decrease in the Bax/Bcl-2 ratio suppresses apoptosis, whereas an increase in the Bcl-2 ratio enhances apoptosis^54^. Therefore, the Bax to Bcl-2 ratio strongly modulates cell survival and apoptosis. In addition, p53 inhibits the anti-apoptotic protein Bcl-2 and promotes the pro-apoptotic protein Bax^55^. Bax and Bcl-2 regulate the mitochondrial outer membrane permeability, stimulate cytochrome C release, activate the caspase cascade, and induce apoptosis^56^. The Fas gene is also important in mediating apoptosis. Based on our findings, increasing Pb concentrations enhanced expression levels of Fas, Bax, p53, caspase-3, and caspase-8 and reduced those of Bcl-2. In addition, the Bax to Bcl-2 ratio was increased. Therefore, Pb can interfere with the levels of multiple apoptotic genes in MSFs. In PC12 cells, Pb triggers apoptosis, accompanied by increased caspase-3 and Bax levels and an associated increase in p53 expression, elevated Bax expression, and reduced Bcl-2 levels^57^. Furthermore, Pb can stimulate Fas gene expression and promote testicular germ cell apoptosis in a dose-dependent manner^58^.

Pb affects the levels of oxidants in MSFs and enhances ROS production; in turn, increased ROS concentration leads to the dysregulation of the cellular lipid bilayer and disrupts Ca^2+^ transport. Pb leads to instability in calcium homeostasis and destroys membrane ion channels by disrupting electron transport and reducing ATP concentration, ultimately leading to cell apoptosis^59^. Pb-induced apoptosis of MSFs also appears to depend on the mitochondria, given that Pb can affect the MMP. Damaged cell membranes are characterized by a decrease in MMP and interference in the balance of molecules such as Bax and Bcl-2^60^. Alterations in the Bax/Bcl-2 ratio can trigger cytochrome C release and caspase activation and, ultimately, cell apoptosis^61^.

However, given that the present study was an in vitro assessment, limitations are inevitable. Considering one such limitation, it should be noted that the results do not necessarily represent real-world effects of Pb on MSFs in vivo, as various other factors affect cell proliferation and apoptosis in vivo. Another limitation of the study is that protein levels of apoptotic markers were not quantified, which can be considered a future research direction.

## Conclusions

In conclusion, Pb induces considerable toxic effects on MSFs, such as decreasing cell survival, accelerating cell morphological alterations, and apoptosis. Treatment with Pb treatment arrested the cell cycle, decreased the MMP, increased the Bax/Bcl-2 ratio, and enhanced caspase-3, -8, and -9 activation, suggesting that Pb can induce MSF apoptosis. These intracellular processes were related to Ca^2+^ overload, suppression of DNA formation, oxidative stress, mitochondrial dysfunction, and altered gene expression. Our findings provide useful regarding the etiology of Pb toxicity in the skin and indicate the undesirable outcomes of Pb exposure in humans. This evidence offers a basis for further studies on the Pb pollutant-based toxicity mechanisms to improve health risk assessments.

## Data Availability

All data generated or analyzed during this study are included in this published article.
